# Interest Groups and Health Facility Regulation – Future Directions for Health Policy and Systems Research: Comment on "What Lies Behind Successful Regulation? A Qualitative Evaluation of Pilot Implementation of Kenya’s Health Facility Inspection Reforms"

**DOI:** 10.34172/ijhpm.2023.7826

**Published:** 2023-04-26

**Authors:** Veena Sriram, Vikash R. Keshri

**Affiliations:** ^1^School of Population and Public Health, School of Public Policy and Global Affairs, University of British Columbia, Vancouver, BC, Canada; ^2^The George Institute for Global Health, Faculty of Medicine and Health, University of New South Wales, Sydney, NSW, Australia; ^3^The George Institute for Global Health India, New Delhi, India

**Keywords:** Interests, Interest Groups, Politics, Health Policy, Regulation

## Abstract

In their paper, Tama and colleagues observe that one key challenge in a pilot, multi-component intervention to strengthen health facility regulation was the reaction from health facility owners and providers to regulatory processes. In this commentary, we propose that future research and action on health facility regulation in low- and middle-income countries (LMICs) contexts adopt an explicit focus on addressing the role of interests and interest groups in health systems ‘hardware’ and ‘software.’ Research on policy processes in LMICs consist of fewer investigations into the political economy of national or sub-national interest groups, such as physician associations or associations of health facility owners. A growing body of literature explores supply-side and demand-side interest groups, power relations within and between these stakeholders, and their advocacy approaches within LMIC health sector policy processes. We posit that such analyses will also help identify facilitators and challenges to implementation and scale-up of similar reforms to health facility regulation.

## Introduction

 In their article exploring a pilot initiative to strengthen health facility regulation in Kenya, Tama and colleagues make a major contribution to current understanding of health sector regulation in low- and middle-income countries (LMICs).^1^ More importantly, the paper presents a novel approach to *reforming* health facility regulation and effectively implementing reform, an area of health sector reform governance has been enormously challenging to address. Tama and colleagues present a multi-component approach to health facility regulation that draws upon both risk-based and responsive regulation, anchored by a joined-up or unified mechanism — the Joint Health Inspections (JHI) — involving eight different regulatory agencies in the health sector. The paper shares results from a robust qualitative study exploring the perspectives of multiple stakeholders involved in the development and implementation of the reform in three counties in Kenya. The findings are promising for scaling-up the approach in Kenya and for stimulating ideas about similar reform in other LMICs, where effective approaches to private health sector regulation have often remained elusive.^2^

 A key challenge expressed in the article is the reaction from health facility owners and providers to decisions taken by the regulatory team, specifically in terms of facility closures. Examples of negative responses include facility owners contesting closure decisions with the Ministry of Health coordinator for the program; facility inspectors indicating personal or professional vulnerability of being identified with closures occurring in the same locality in which they live; and facility owners using connections with “*big people in the county offices”*^1^ to continue facility operation despite low scores and a closure decision. The mechanism of top-down, bureaucratic control has also proven to be highly problematic, and there is an urgent need for integrated, holistic approaches to optimizing health facilities regulation.

 A small but growing body of literature on health sector regulation in LMICs indicates a deeper undercurrent of stakeholders acting in their interests to push against ‘unfavorable’ decisions at multiple stages of policy process, and these same stakeholders often organize themselves to threaten the expansion of those decisions across contexts. In this commentary, we propose that future research and action on health facility regulation in LMICs contexts adopt an explicit focus on the role of interests and interest groups in health systems ‘hardware’ (formal structures such as legislation and organizations) and ‘software’ (ideas, values and norms) issues in the context of proposed reforms. In addition to providing a more nuanced analysis of stakeholder perspectives, we posit that such analyses will also help identify facilitators — and importantly, challenges — to implementation and scale-up. In this commentary, we begin by situating interest groups in research on health sector regulation in LMICs. We then summarize key insights from existing studies on the politics of interest groups in health sector regulation. We conclude by linking these ideas to the insights presented in the Tama et al paper.

## The Role of Interest Groups in Health Sector Regulation in LMICs – a Neglected Aspect of Health Policy and Systems Research

 Research on the politics of health policy processes in LMICs has been steadily growing in recent years, providing rich and valuable insights on the facilitators and barriers to policy change in these contexts.^3^ Yet, the role of domestic or national stakeholders outside of government and the nonprofit sector, such as interest groups representing doctors, hospitals, businesses, civil society, etc, remains limited in the current knowledge base. Interests are defined as “the advantages and disadvantages that implementation of the policy may bring to a stakeholder or [their] organization.”^4^ Interest groups by extension, are those organizations that represent these interests of specific stakeholders to government. As defined by Yoho, “interest groups are comprised of actual organizations, rather than multiple persons who are unorganized; they attempt to influence government; they are not themselves government agencies, however; and neither are they political parties.”^5^

 Analyses of interest groups in the health sector have led to important insights, such as the role of interest groups in facilitating or blocking policies that would address health inequities, access disparities or challenges faced by health workers, including actions taken by those representing the medical profession or the hospital industry.^6,7^ Actions taken by interest groups must not be seen in a binary of promoting or threatening health systems strengthening or goals around reducing health inequities; rather, interest groups represent diverse constituencies with diverse goals, strategies and coalition partners. Global health research consists of fewer investigations into the political economy of national or sub-national interest groups, specifically, how these interest groups use their power and resources to shape health policy in LMICs. Additionally, limited research explores the capacity of governments in engaging with interest groups, and the relationships and interactions between governments and interest groups.

 This gap extends to research on regulation, an area where interest group politics is critical to grapple with due to the likelihood of ‘regulatory capture’ – “the process through which special interests affect state intervention in any of its forms” threatening the objectives of a regulatory system.^8^ Examples of regulatory capture in the health sector include collusion between regulatory agencies and professional associations representing the target constituency of a particular regulatory agency or pharmaceutical companies seeking to weaken regulatory policy around particular drugs.^9^ The limited set of studies that investigate the politics of regulatory systems suggest that these processes are strongly influenced by the politics of interest groups. For example, longstanding efforts to regulate the private health sector in India — a sector of immense complexity and diversity — through the Clinical Establishments Act have repeatedly faced intense opposition from interest groups such as those representing doctors and hospitals.^10^ A multi-country study in Cambodia, Indonesia, and Pakistan examining the impact of interactions between policy-makers, health providers and pharmaceutical companies on regulatory policy in the pharmaceutical sector found serious conflict of interest concerns with regards to the actions taken across the policy cycle.^9^ Four types of connections between pharmaceutical companies and providers were identified — financial, political, social, and familial ties.^9^ Building on these studies, we propose that researchers exploring regulatory innovation in the health sector more explicitly examine the role of interest groups in the processes to establish these systems and to ensure effective implementation.

## The Politics of Interest Groups in Health Sector Regulation

 As the JHI potentially expands in Kenya and other countries, decision-makers have an expansive task in generating consensus and cohesion across disparate regulatory agencies (an impressive aspect of the JHI process given the often-entrenched nature of each regulatory system). In addition, decision-makers must pay close attention to the ways in which interest groups representing key stakeholders in the process, shape development, implementation and scale-up processes. These interest groups are heterogeneous in many contexts, with for example different organizations representing corporate hospitals and small hospitals, respectively, and might therefore be variously advantaged or disadvantaged by policy decisions. Interest groups are also highly effective at forging coalitions depending on their interests and social positioning (ie, knowledge, class, educational and familiar connections, access to powerful actors through patient base, etc). For example, interest groups may formally coalesce to push back on regulation, particularly as it pertains to criteria that pertains to closures, or to grievance redressal processes. There is currently limited knowledge on how these interest groups organize in LMIC settings, but available evidence suggests that these groups are highly strategic and work to access top-levels of government through personal networks, industrial action, etc.^10,11^ Interest groups also actively utilize resources to move policy debates to the (often arduous and complex) judicial system to safeguard their interests.

 Interest groups represent supply-side or demand-side constituents in the case of health facility regulation. Supply-side stakeholders consist of health facility owners or health workers, while demand-side stakeholders would include patients’ groups, patients’ rights organizations, or larger civil society organizations. There are some rich examples of “citizen-led accountability initiatives”^12^ from LMICs, showcasing their importance in demanding justice for malpractice, and in seeking more accountability from health facilities. In the case of one Southern Indian state, civil society vociferously argued that private health sector regulation should be “pro-patient,” opposing the demands of health facility owners and physician interest groups to constrain grievance redressal processes and limit punitive actions for failing to conform to regulations.^13^ However, in health sector reform processes, the role of patients’ rights organizations is not well researched, particularly in LMICs. Therefore, the relative power of ‘demand side’ and ‘supply side’ interest groups must be carefully examined, particularly in contexts where the later groups representing health facilities or physicians wield considerable power. Conversely, patients’ rights organizations lack representation and voice in policy processes, often due to broader societal factors of marginalization and disenfranchisement.^13^ Such power relations within and across constituencies are therefore critical to explore further.

 Tama and colleagues correctly note that “it is essential to listen to the voices of the actors directly involved, to understand their views, perceptions and experiences.”^1^ Interest groups would likely play a major role in voicing those concerns at multiple stages of the policy process. For example, interest groups representing single-doctor practices, clinics or smaller scale hospitals might discuss challenges pertaining to meeting standards when compared with better resourced facilities, an issue noted in the commentary, and a concern emerging in similar setting like India. That said, the same interest groups representing stakeholders impacted by regulatory processes must be carefully engaged with to deal with unfair influence, co-opt or disrupt regulatory processes (referred to as ‘managing’ in stakeholder analyses).

 Informally, as noted in the study, impacted stakeholders may tap into personal and professional networks, formed through factors such as political linkages, geographic location, familial or educational connections, etc to contest closures and other aspects of regulation. Formal connections (for example, interest groups or government) and informal networks (personal and social connection) are crucial to understand in order to analyze the impact of regulatory policy on key stakeholders. As put forward by Baez Carmago and Koechlin, formal networks can be used as camouflage for informality.^14^ They write, “informal practices of co-optation and control take place beneath a facade of commitment to formal procedures,”^14^ which could be extended to the ways in which interest groups and their constituencies might influence policy formulation or implementation.

## Using Health Systems “Hardware and Software” to Manage the Role of Interest Groups in Health Facility Regulation

 Tama and colleagues rightly identified the ‘software’ factors which played an important role in the success of the JHI. Software, defined as “the ideas and interests, values and norms, and affinities and power that guide actions and underpin the relationships among system actors and elements”^15^ has long been recognized as a critical aspect of health sector regulation, but few interventions have explicitly addressed these factors. Regulatory approaches till date have often focused on health systems ‘hardware,’ the formal structures that might include legislation, organization and formal governance processes.^15^ Moving forward, nuanced analyses around prevailing systemshardware and softwarecan be an important framework to research, analyze and propose policy changes for effective regulation of health facilities in LMICs.

 Interest groups interact with health systems hardware and software in two ways. Health systems hardware, including institutional frameworks for interest group involvement in policy processes, determines the formal extent to which interest groups may engage in policy-making and their power within the system. For example, interest groups in certain contexts are legally permitted to make campaign contributions to political campaigns. The members of interest groups are often embedded in systems hardware through policy formulation and implementation processes.^6^ Health systems software refers to actor relationships, power dynamics and political processes, and helps explain the ways in which interest groups shape, influence or block policy processes at one or more policy stages in order to achieve their goals. For example, certain interest groups might have high levels of power and influence within the health sector, due to their embeddedness in institutional frameworks, and also due to their ‘soft power’ by virtue of social positioning or professional hierarchies. Health systems hardware and software also have strong hues of colonial systems in the case of many LMICs. For example, the evolution of physicians as elite social groups with extensive power and political networks have resulted in their disproportionate influence over health sector regulation.

 There are major implications of the interactions between interest groups and hardware and software aspects of health system regulation. For example, interest groups might seek to weaken appeals processes in the policy formulation stage or take shortcuts around formal appeals processes and rely on personal networks to contest facility closures, as seen in the JHI example in Kenya. Additionally, it is important to ensure that engaging interest groups in policy processes does not allow them to co-opt processes to suit their interests by relying on aspects of health systems ‘software.’ Ultimately, decision-makers seeking to expand approaches such as JHI should pay careful attention to how health system hardware and software interact with interest groups.

## Conclusion

 In this commentary, we have sought to highlight the key role of interests and interest groups in heath sector regulation and propose that future research and action adopt an explicit focus on these factors. The JHI is an exciting opportunity to transform the regulation of health facilities in Kenya and LMICs more broadly.^1^ We posit that an explicit focus on interest groups, including demand- and supply-side stakeholders, their politics, and their role on health system hardware and software will proactively surface potential partnerships, opportunities and also challenges at multiple stages of the policy cycle. This approach will also identify the strategies for implementation and reform. Addressing these aspects of heath sector regulation will potentially enable more successful implementation and scale-up of reform efforts, contribute to strengthened health systems and ultimately, improve population health.

## Ethical issues

 Not applicable.

## Competing interests

 Authors declare that they have no competing interests.

## Authors’ contributions

**Conceptualization:** Veena Sriram.

**Writing–original draft:** Veena Sriram and Vikash R. Keshri.

**Writing–review & editing: **Veena Sriram and Vikash R. Keshri.
